# Quantification of somatic mutation flow across individual cell division events by lineage sequencing

**DOI:** 10.1101/gr.238543.118

**Published:** 2018-12

**Authors:** Yehuda Brody, Robert J. Kimmerling, Yosef E. Maruvka, David Benjamin, Juniper J. Elacqua, Nicholas J. Haradhvala, Jaegil Kim, Kent W. Mouw, Kristjana Frangaj, Amnon Koren, Gad Getz, Scott R. Manalis, Paul C. Blainey

**Affiliations:** 1 Klarman Cell Observatory, Broad Institute of MIT and Harvard, Cambridge, Massachusetts 02142, USA;; 2 Broad Institute of MIT and Harvard, Cambridge, Massachusetts 02142, USA;; 3 MIT Department of Biological Engineering, Cambridge, Massachusetts 02139, USA;; 4 Koch Institute for Integrative Cancer Research, MIT, Cambridge, Massachusetts 02139, USA;; 5 MGH Cancer Center and Department of Pathology, Boston, Massachusetts 02114, USA;; 6 Harvard Medical School, Boston, Massachusetts 02115, USA;; 7 Department of Radiation Oncology, Brigham and Women's Hospital, Dana-Farber Cancer Institute, Boston, Massachusetts 02115, USA;; 8 Department of Molecular Biology and Genetics, Cornell University, Ithaca, New York 14853, USA

## Abstract

Mutation data reveal the dynamic equilibrium between DNA damage and repair processes in cells and are indispensable to the understanding of age-related diseases, tumor evolution, and the acquisition of drug resistance. However, available genome-wide methods have a limited ability to resolve rare somatic variants and the relationships between these variants. Here, we present lineage sequencing, a new genome sequencing approach that enables somatic event reconstruction by providing quality somatic mutation call sets with resolution as high as the single-cell level in subject lineages. Lineage sequencing entails sampling single cells from a population and sequencing subclonal sample sets derived from these cells such that knowledge of relationships among the cells can be used to jointly call variants across the sample set. This approach integrates data from multiple sequence libraries to support each variant and precisely assigns mutations to lineage segments. We applied lineage sequencing to a human colon cancer cell line with a DNA polymerase epsilon (*POLE*) proofreading deficiency (HT115) and a human retinal epithelial cell line immortalized by constitutive telomerase expression (RPE1). Cells were cultured under continuous observation to link observed single-cell phenotypes with single-cell mutation data. The high sensitivity, specificity, and resolution of the data provide a unique opportunity for quantitative analysis of variation in mutation rate, spectrum, and correlations among variants. Our data show that mutations arrive with nonuniform probability across sublineages and that DNA lesion dynamics may cause strong correlations between certain mutations.

Somatic mutations are implicated in age-related diseases including cancer, neurodegeneration, and organ failure in humans (Rossi et al. 2008; Martincorena and Campbell 2015; Fernández et al. 2016; Vijg et al. 2017; Lodato et al. 2018). Understanding the intrinsic and extrinsic factors that contribute to mutagenesis (including mutation-inducing cancer therapies) is currently a top priority in cancer prevention and treatment (Wu et al. 2016; Drost et al. 2017; Vijg et al. 2017; Knijnenburg et al. 2018; Seluanov et al. 2018). However, available methods for somatic mutation detection are far from satisfactory, limiting our progress in understanding how mutations accumulate in cells and impact health (Gawad et al. 2016; Baslan and Hicks 2017; Zou et al. 2018). Despite the complexity of nucleoside chemistry, the wide range of DNA lesions characterized in humans, and the large variety of DNA replication and repair enzymes, only 30 distinct somatic mutation signatures have been defined from human tumor sequencing, and the etiology of more than half remains unknown (Petljak and Alexandrov 2016). Analysis of quantitative, high-resolution, and unbiased somatic mutation data has the potential to link observed mutation spectra with molecular mechanisms. However, the distributed and asynchronous nature of mutations in somatic cells makes these mutations difficult to detect and accurately quantify. Specifically, published rates of somatic mutation vary widely from 10^−11^ to 10^−7^ single nucleotide variants (SNVs) per base pair per cell division (SNV/bp/division) (Lynch 2010; Holstege et al. 2014; Li et al. 2014; Blokzijl et al. 2016; Ju et al. 2017; Milholland et al. 2017) due to differences across cell types and reliance on uncertain cell division rate estimates (Tomasetti et al. 2017). Without strong selection such as that occurring in tumor development (McGranahan and Swanton 2017), these somatic mutation rates result in variant allele frequencies lower than can be reliably detected in bulk samples using standard sequencing approaches, which produce consensus false positive SNV error rates of 10^−5^ to 10^−4^ per nucleotide (Reumers et al. 2011; Schmitt et al. 2012). Thus, there is a need for quantitative and accurate genome-wide somatic mutation analyses that are unbiased by positive or negative selection (Levy et al. 2015).

Available approaches for somatic variant analysis have provided valuable insights but also exhibit technical limitations that compromise quantitative mutation analysis. Extremely rare alleles in bulk samples can be detected by molecular consensus sequencing, although a requirement for ultradeep coverage makes genome-wide analysis challenging (Schmitt et al. 2012; Martincorena et al. 2015; Merkle et al. 2017; Salk et al. 2018) and such approaches do not directly report on intra-lineage structures or dynamics. To enrich samples for particular somatic variants, single cells can be isolated and cloned or processed for sequencing directly. These cells or clones are typically separated by a large and unknown number of cell division events, which contribute to uncertainty in mutation rate calculations and severely limit the power to determine exactly when the mutations arose within a lineage and correlations among the mutations (Blokzijl et al. 2016; Szikriszt et al. 2016; Assaf et al. 2017; Drost et al. 2017; Milholland et al. 2017; Pfeiffer et al. 2018). Direct single-cell methods do not require live cells or suffer from a selection bias against slower-growing or nongrowing cells. Despite recent advances, however, these methods remain compromised in the sensitivity and accuracy of variant detection compared with bulk approaches (Lodato et al. 2015; Gawad et al. 2016; Xu et al. 2016; Chen et al. 2017). Mutation accumulation experiments that run for hundreds of generations or more to enrich lineages with large numbers of mutations are widely used for fast-growing bacterial cells (Tenaillon et al. 2016) but are impractical for mammalian cells. Other approaches for measuring mutation rates in vitro target specific genomic loci, which can strongly bias estimates of genome-wide mutation rates and spectra (Araten et al. 2005). Thus, no previous approach has combined (1) the ability to identify groups of variants that arise during the replication of an individual cell, (2) high accuracy and sensitivity for genome-wide somatic SNV detection, and (3) minimal positive and negative mutation selection biases.

Here, we introduce lineage sequencing (Fig. 1) and its application to clonal cell populations cultured in vitro. In lineage sequencing, one collects specific cells representing different lineages, subclones these cells (or otherwise amplifies the subject genomes), and shotgun sequences each subclonal population. De novo variants arising during the growth of the original population are shared between subclones and detected with high sensitivity and specificity by identifying somatic variants across the subclones that are consistent with the estimated lineage structure of the population. In regions of the lineage that are well-covered by subclones, the origin of variants can be pinpointed to an individual cell division event.

**Figure 1.**
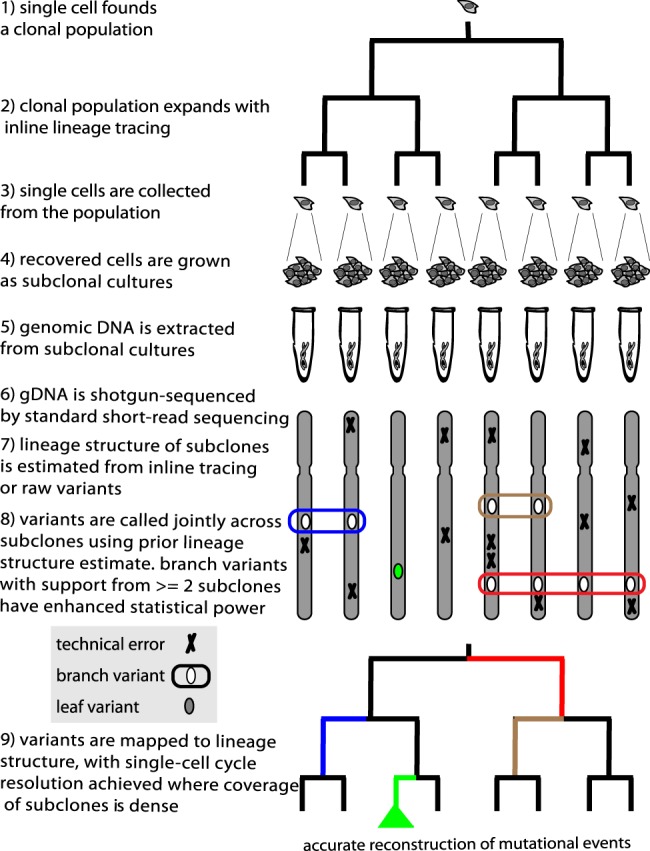
Lineage sequencing concept and implementation. Overview of the lineage sequencing concept. Numbering indicates key conceptual and implementation steps. Single cells are sampled from a clonal population and sequenced (steps 1–6; in this study, subclonal culture was used to produce enough genomic DNA for PCR-free shotgun sequence library construction). Crucially, a prior estimate of the population lineage structure (step 7; either from single-cell tracking by time-lapse imaging or from raw SNV calls) was used to identify novel somatic variants in a joint analysis of the sequence libraries (step 8). This use of the lineage information enables all the sequence libraries to provide statistical support for somatic “branch variants,” enhancing the sensitivity and specificity of somatic variant identification (for example, the schematically indicated “red variant” is supported by presence of an SNV in four sequence data sets from one sublineage and the absence of this SNV in four additional sequence data sets from the other sublineage). Where the coverage by sampled sublineages (via subclones in this study) is high, the mutations that appeared during the lineage experiment can be mapped with single-cell resolution onto the lineage (step 9; e.g., blue, red, tan segments in the dendrogram at bottom). We term mutations occurring in the last round of cell division events “leaf variants,” which by definition can be supported only by a single sequence data set (e.g., green segment in the dendrogram at bottom). Leaf variants can also be analyzed but do not benefit from the enhanced statistical power that supports branch variants and thus cannot be reliably assigned to specific cellular events.

In this proof-of-concept demonstration, we precisely controlled cells in the population using a microfluidic device (Kimmerling et al. 2016) and carried out time-lapse microscopy to track cells and provide independent knowledge of the relationships between subclones (Supplemental Fig. S1; Supplemental Movie MS1). In this work, we apply the device for culturing and manipulating single cells in preparation for assessment of the genomic changes in individual sublineages. In contrast, our previous work using this and other microfluidic device classes focused on direct analysis of RNA to study transcriptional heterogeneity (Kimmerling et al. 2016) and integrated sample preparation for sequence library construction (Kim et al. 2017).

Initially, we used the lineage structure generated from the microscopic tracking to search for somatic variants (optical tracking → lineage → called variants). Subsequently, in order to test the accuracy and sensitivity of lineage sequencing in the absence of cell tracking results, we blinded ourselves to the imaging data and generated a prior estimate of the lineage structure using only the sequence data (raw variants → lineage → called variants). This sequencing-only implementation resulted in equivalent somatic variant detection performance but does not allow observed single-cell phenotypes to be linked with single-cell somatic events. By resolving the lineage at high cellular resolution, lineage sequencing is able to probe intra-lineage heterogeneity of mutational processes. For example, we apply lineage sequencing to compare mutation rates across different lineage segments, to identify statistical dependencies between mutations, and to suggest mechanistic links between certain mutation sets.

## Results

### Application of lineage sequencing to cultured human cells

To demonstrate the lineage sequencing concept (Fig. 1), we selected two cell lines expected to exhibit different mutation rates and spectra. The human colon carcinoma cell line HT115 possesses a missense mutation in one copy of the DNA polymerase epsilon (*POLE*) gene, specifically a V411L mutation in the exonuclease domain which has been associated with hypermutated cancer phenotypes (Barretina et al. 2012; Forbes et al. 2015). POLE exonuclease (‘proofreading’) deficiency has been identified in a subset of human tumors and results in high mutation burdens and a unique mutation signature (Albertson et al. 2009; Lange et al. 2011; Shinbrot et al. 2014; Haradhvala et al. 2018). The human retinal pigmented epithelium cell line RPE1 was immortalized by telomerase reverse transcriptase (*TERT*) overexpression and has not been annotated as exhibiting a mutator phenotype (Bodnar et al. 1998). For each cell line, we grew lineages to five or six generations starting from a single founding cell. This culture step was carried out in a custom microfluidic device able to track and manipulate small populations of cells arising from a single founding cell (Supplemental Fig. S1; Supplemental Movie MS1). The same device was previously used in a different study to measure transcriptional heterogeneity among related cells with RNA-seq (Kimmerling et al. 2016). The device contains an array of hydrodynamic trap structures that enable long-term growth with the entire population under continuous observation by time-lapse imaging. The device is also configured to release cells one at a time, a capability we utilized to collect cells from the population and establish subclonal cultures representing individual lineages. From our time-lapse data, we were able to measure the inter-division times for all cells in the device. We extracted genomic DNA from subclones and prepared PCR-free shotgun sequence libraries, which were sequenced to 35-fold coverage (Methods). The sampled lineages show a similar distribution of inter-division times compared with the overall distribution, showing that there is no obvious bias in our results toward cells/lineages with any particular inter-division time (Supplemental Fig. S2).

### Lineage structure information can be utilized in variant calling across multiple data sets

**Figure 2.**
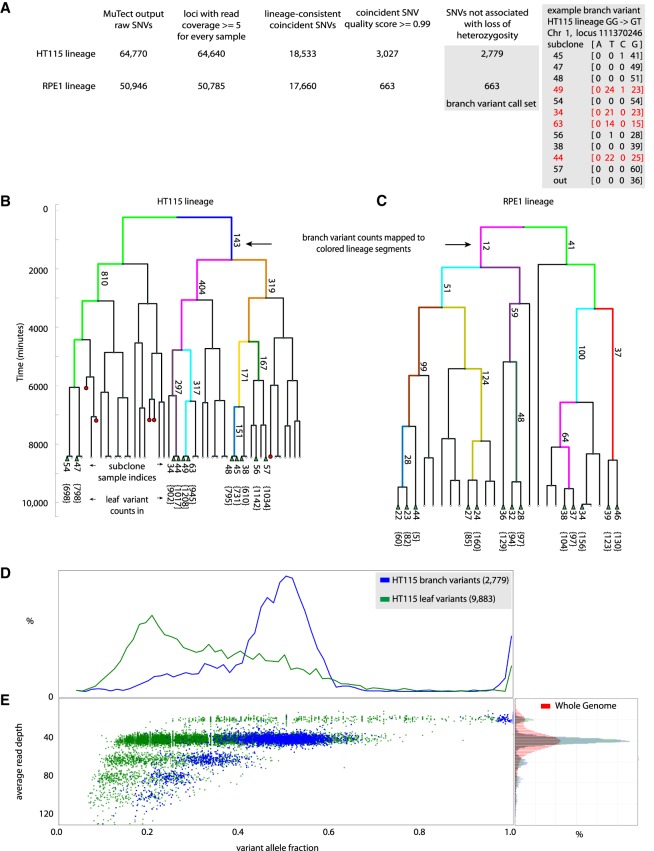
HT115 and RPE1 lineage sequencing experiments by the “optical tracking → lineage → called variants” approach. (*A*) Scheme of the analysis pipeline for identifying branch variant SNVs by the “optical tracking → lineage → called variants” approach. “Branch variants” are SNVs that occur at the same locus in two or more (but not all) subclones and are consistent with prior lineage information. Variant counts at different stages of the informatics filtering steps used to identify high-quality lineage structure concordant branch variants are shown for the HT115 and RPE1 lineage sequencing experiments. Detail on an example HT115 GG → GT branch variant is shown. Allele counts from sequence reads at Chromosome 1 diploid locus 111370246 are shown. Four subclone sequence libraries (subclone indices 49, 34, 63, and 44, marked in red) show about half the reads indicating a variant T allele, where all the other subclones support only the reference G alleles at this locus. This G → T SNV is scored as one of 404 branch variants that appeared within the two cell cycles represented by the pink segment on the *right*-hand side of the dendrogram representing the HT115 lineage experiment in *B*. (*B*,*C*) Dendrograms representing the HT115 and RPE1 lineage experiments; red circles mark time points where cells died during the lineage development and were not available for recovery from the device. The green triangles in the *bottom* of the dendrogram represent cells that were recovered, subcloned, and sequenced. Dendrograms are annotated with the count of “branch variants” for resolved lineage segments (some segments are resolved to individual cell cycles). Every sequenced subclone is annotated with its index number and the count of “leaf variants” for each sequenced subclone (at *bottom*). “Leaf variants” are SNVs that are supported by only one subclone and likely represent variants that arose during or after the last generation of the lineage experiment. The *x*-axis of the dendrogram only relates to linkage of the subclones. The *y*-axis of the dendrogram represents the culture time course, with each cell division event observed by time-lapse imaging marked by a branch point in the dendrogram. Single cells were recovered for subculture from the HT115 lineage after 141 h, while cells were recovered from the RPE1 lineage after 168 h. (*D*) HT115 branch variants are clonal. Histogram of allele fraction for detected variants. Comparison between branch variants (mutations occurring during lineage formation up to the last cell division) and leaf variants (mutations occurring within or subsequent to the last cell division event in the lineage). Branch variant SNVs show a bimodal allele fraction distribution peaked at 0.5 and 1.0 as expected for the measured ploidy (copy number variation [CNV] analysis) at variant loci in this mostly diploid cell line. In contrast, subclonal mutations appear in the leaf variant group and show an allele fraction distribution peaked well below 0.5 as the variant caller attempts to balance sensitivity for low allele fraction variants with false-positive detections without the enhanced performance available for branch variants. (*E*) *Left* panel: scatter plot of variants; average read depth versus allele fraction; branch variants (blue) and leaf variants (green). The branch variant read depth is tightly correlated with the variant allele fraction in accordance with clonal mutations. The leaf variants include many subclonal variants that blend with technical noise at low variant allele fractions. *Right* panel: normalized histogram of read coverage depth for HT115 lineage; whole-genome (red), called branch and leaf variants (blue and green).

In order to produce a list of provisional SNVs for each lineage experiment, sequence data from all pairs of subclones were analyzed with MuTect1 (Cibulskis et al. 2013) using the hg19 reference (Genome Reference Consortium GRCh37). SNVs that arose de novo during the lineage experiment were identified by filtering for groups of SNVs produced by the primary variant calling analysis that occurred at the same locus in two or more (but not all) subclones. Such groups of matching SNVs that coincide at the same genomic locus in multiple subclones putatively represent de novo somatic mutations that occurred during generations 1–5 in the lineage experiments. We term these SNVs “branch variants” (Fig. 2A–C). In contrast, SNVs shared by all subclones were most likely present in the founding cell.

SNVs appearing in only one subclone are termed “leaf variants” and likely represent variants that either appeared in the last round of cell division, appeared early in subclonal culture (or later in culture if strongly selected), or represent technical errors in sequencing or variant calling. Variants arising during subclonal culture are excluded from the branch variant call set, which only accepts variants present in at least two subclones. Using the branch variants, which represent de novo somatic mutations that appeared in generations 1–5 of the lineage experiments, we quantitatively reconstructed mutation events and the flow of mutations through the lineages (Fig. 2B and Supplemental Table S2 for HT115; Fig. 2C and Supplemental Table S3 for RPE1). Branch variants are expected to appear as fully penetrant clonal variants in the affected subclonal populations because they occur before the subcloning step. In HT115, such coincident SNV sets constituting branch variants were enriched at allele fractions close to 0.5, as expected for clonal mutations in a predominantly diploid genome (Fig. 2D; corresponding RPE1 allelic fraction results are shown in Supplemental Fig. S3). The allele fraction distribution of clonal branch variants is concordant with the copy number variation analysis for both cell lines (Fig. 2E; Supplemental Figs. S3B, S4).

In contrast, noncoincident SNVs representing variants arising within or after the last (sixth) generation of the HT115 lineage—the leaf variants—had to be identified within individual samples. The leaf variants showed an allele fraction distribution distinct from the branch variants with most values lower than 0.5 and range down to uncertain instances of candidate variants with low allele fraction that are filtered out by the variant caller (Fig. 2D,E and Supplemental Fig. S3 for RPE1).

The knowledge that branch variants must be clonal is valuable in variant detection. For example, we can easily segment mutations according to the copy number determined at each genomic locus from the read coverage depth in our 35× PCR-free data since variant alleles are known to be clonal. Coverage to 35× performs well for branch variant calling since the reduced average read depth at lower ploidy sites is compensated for by the higher allele fraction and the low coverage dispersion of our PCR-free data. Our ability to apply relaxed thresholds in calling branch variants with a low chance of false-positive detections makes branch variant calling more sensitive and quantitative than standard approaches.

Leaf variants in our data include subclonal variants, and their detection is fraught with challenging tradeoffs in read depth and variant allele fraction cutoffs (Fig. 2E for HT115; Supplemental Fig. S3B for RPE1).

To test how these tradeoffs are realized across different variant callers, we reran the analysis with a different variant caller, Strelka (Saunders et al. 2012). The Strelka and MuTect1 results for branch variants were highly similar, with Strelka making up to 3% more branch variant calls but recapturing better than 99% of MuTect1 calls, reflecting the high accuracy of both branch variant call sets (Supplemental Fig. S5). There was lower but still extensive overlap in the Strelka and MuTect1 calls from the leaf variant data set (80%–90%) as expected due to the lower certainty of the leaf variant calls and the expected variance in lower-confidence calls across algorithms (Supplemental Fig. S5; Cai et al. 2016). To further test the robustness of our results, we also called variants with MuTect2 against an updated reference genome hg38 (GRCh38). We found that the call sets overlap extensively with the MuTect1 calls made against hg19 (GRCh37) (branch variants: >96%; leaf variants: >93%) (Supplemental Fig. S5C).

### Lineage sequencing recapitulates mutational characteristics of POLE proofreading deficiency

**Figure 3.**
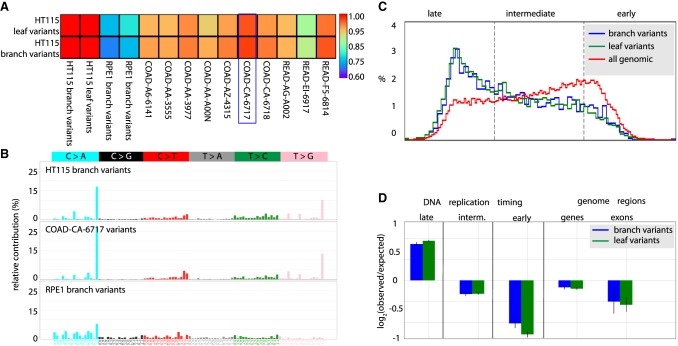
Analysis of mutation patterns in human colon carcinoma epithelial cell line HT115. HT115 shows POLE proofreading deficiency that matches previously published bulk *POLE* mutant colon tumor sample data. (*A*) Heat map showing the cosine similarity scores of comparisons of whole-genome HT115 variant SNV mutation spectra with whole-genome spectra from RPE1 samples and published data sets from *POLE* mutant tumor samples (Cancer Genome Atlas (TCGA), dbGAP: phs000178.v1.p1; sample annotations in Supplemental Table S1). The blue rectangle denotes the most similar tumor sample (COAD-CA-6717). (*B*) Comparison of detailed mutation spectra of all base substitutions observed in HT115 and RPE1 cell line branch variants and in the COAD-CA-6717 TCGA sample. HT115 and COAD-CA-6717 show highly similar spectra that differ from the RPE1 spectrum. (*C*) Distribution of DNA replication timing for all genomic positions and the somatic SNV branch variants and leaf variants (blue and green, respectively) from the HT115 cell line. Both the branch and leaf variant sets show the expected enrichment in late-replicating regions and depletion in early-replicating regions versus the background distribution of replication timing at all genomic loci (red). (*D*) Quantification of the enrichment and depletion of SNVs in the indicated categories. SNVs are enriched in the late-replicating regions while SNVs are depleted in RefSeq genic regions and further depleted in RefSeq exons (*P* < 0.01) in both the branch and leaf variant SNV sets.

Mutations are driven by the biochemistry of nucleotides, DNA, and their metabolism, including synthesis and repair. Each mutagenic pathway has particular characteristics and results in a distinct mutation spectrum (Alexandrov et al. 2013a,b). As a result, observed mutation spectra provide clues about the mutational processes operating in cells. The spectrum of mutations caused by POLE proofreading deficiency has been previously characterized (Shinbrot et al. 2014). We compared the mutation spectrum of our aggregated HT115 branch variant SNV call set with mutation spectra derived from tumor-normal whole-genome sequencing of group A *POLE* colon tumors (samples with mutant *POLE* but not mutant *POLD1*) (Shinbrot et al. 2014) generated by the TCGA Research Network and found high similarity (Fig. 3A). The highest similarity to our HT115 spectrum was found with a particular colorectal tumor sample annotated with stop codon (R1371*) and a missense mutation L1235I in the *POLE* gene (cosine similarity = 0.97 ± 0.01) (Fig. 3B; Supplemental Table S1). The RPE1 spectrum differs markedly from the HT115 spectrum (cosine similarity = 0.67 ± 0.015) and the *POLE* mutant clinical tumor spectra (Figure 3A) and COSMIC mutation signature analysis (Supplemental Fig. S6).

The activity of the DNA mismatch repair (MMR) pathway is known to be coordinated with DNA replication and to be most active during S phase, particularly in euchromatic early-replicating regions (Kunkel and Erie 2015; Supek and Lehner 2015). Using high-resolution genomic replication timing data, we compared the frequency of SNVs and replication timing across the genome (HT115 replication timing, Supplemental Table S4). SNVs were markedly suppressed in early-replicating regions relative to late-replicating regions (HT115 in Fig. 3C,D and RPE1 in Supplemental Fig. S7). We also observed significantly fewer SNVs in genic regions than in inter-genic regions and an even higher bias against variants in exons (Fig. 3D; Supplemental Fig. S5; Dulak et al. 2013; Li et al. 2015; Frigola et al. 2017). These effects were observed in both the branch and leaf variant call sets and are likely attributable to differential epigenomic status and repair efficacy across different classes of genomic loci (Schwartz et al. 2009; Huff et al. 2010; Li et al. 2013; Frigola et al. 2017; Smith et al. 2017). Such local variation in mutation rates across the genome underscores the need for mutation rates to be assessed on a genome-wide basis, as targeted approaches are likely to be biased by sampling any particular subset of genomic loci.

*POLE* mutations are also associated with a particular type of strand asymmetry called replication-class (R-class) asymmetry (Haradhvala et al. 2016), which arises due to *POLE*’s specific role in synthesis and proofreading of the leading strand during DNA replication and the stereotyped locations of replication origins in much of the human genome. *POLE*-driven mutations appear in these regions in a polarized fashion with respect to the two DNA strands. For example, we expect a high proportion of C > A relative to G > T in the DNA strand being synthesized as the leading strand (C > A in left replicating regions and G > T in right replicating regions relative to the genomic reference). Indeed, we observed the predicted *POLE* R-class asymmetry among HT115 branch variant SNVs at the same level previously quantified in TCGA *POLE* mutant samples (Supplemental Fig. S8; Haradhvala et al. 2016).

### Lineage sequencing without optical cell tracking

**Figure 4.**
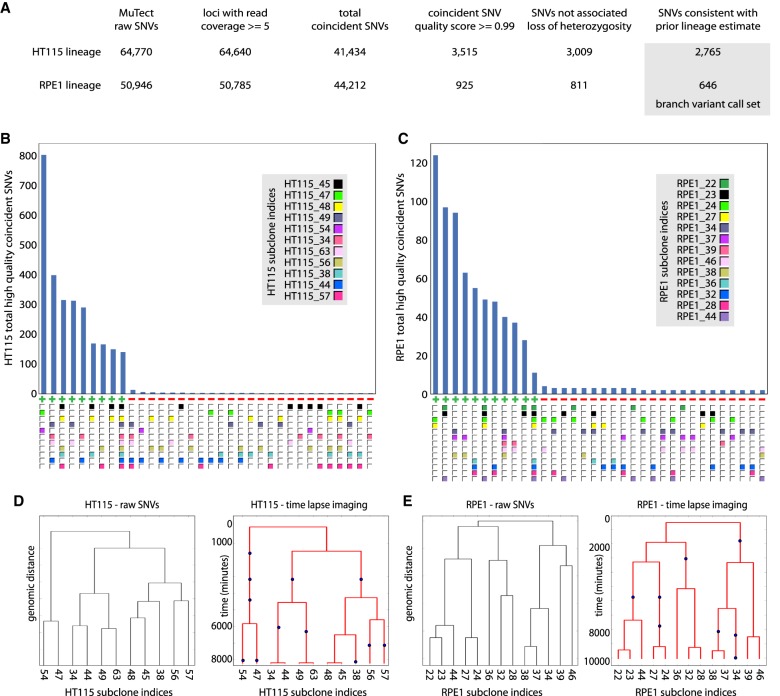
Accuracy and sensitivity of lineage sequencing without microscopic tracking. (*A*) Scheme of the analysis pipeline for identifying branch variants by the “raw variants → lineage → called variants” approach. Variant counts at different stages of the informatics filtering steps used to identify high-quality lineage structure concordant branch variants are shown for the HT115 and RPE1 lineage sequencing experiments. The pipeline is similar to the “optical tracking → lineage → variants pipeline” (Fig. 2A) except that lineage information is incorporated later, separately from SNV coincidence, and the source of the prior lineage estimate is analysis of raw SNVs (see *B* and *C*) rather than time-lapse imaging. (*B*,*C*) Histogram of the number of high-quality coincident SNVs for each set of subclones in which such variants occurred for the HT115 (*B*) and the RPE1 (*C*) data sets. At *bottom*, each cluster is marked as consistent (+) or inconsistent (−), with the lineage structure indicated by the time-lapse imaging. For each cell line, the group of subclone sets with high frequencies of these SNVs are both internally self-consistent and consistent with the independent time-lapse imaging data. (*D*,*E*) Comparison between dendrograms representing lineages based on genomic distance among subclone pairs and the time-lapse imaging data (only subclones that were cultured and sequenced are represented); HT115 (*D*) and RPE1 (*E*). The dendrograms based on genomic distance and time-lapse imaging indicate the same connectivity between subclones, the information relevant to joint variant calling in lineage sequencing, but have different branch lengths and are missing several internal cell divisions. The blue dots in the time-lapse imaging dendrogram represent cell division events that are not independently available from the sequence data. The dendrograms based on time-lapse imaging have a *y*-axis with units of minutes.

We tested whether lineage structures could be estimated from the genomic data alone by blinding ourselves to the time-lapse imaging data from the HT115 and RPE1 experiments. We first filtered the raw SNV calls from MuTect1 to identify coincident SNV calls (Fig. 4A). We then counted the number of coincident SNVs for each set of subclones in which coincident SNVs occurred and plotted the ranked counts for the HT115 and the RPE1 experiments (Fig. 4B,C). A small number of subclone sets contained high counts of coincident SNVs (HT115: >100; RPE1: >10), while other sets contained very low counts (HT115: <10; RPE1: <5). The groups of subclones with high frequencies of coincident SNVs comprised a consistent set of relationships that predicted a single lineage structure for each experiment (Fig. 4D,E, dendrograms plotted based on total set of high quality coincident SNVs). Using these lineage structure estimates, we then recovered nearly the same sets of branch variant calls identified earlier using the time-lapse imaging data to track cells (SNV recovery versus the time-lapse imaging-informed approach: HT115, 99.4%; RPE1, 97.1%). However, time-lapse imaging adds the ability to detect the accurate number of cell divisions between subsets of cells irrespective of mutation rate (Fig. 4D,E). Additionally, for cases with a low number of mutations per cell cycle or dynamic changes in mutagenesis, time-lapse imaging would be necessary for reliable identification of subclone relationships.

### Lineage sequencing is accurate and sensitive

Lineage sequencing also allows data quality testing by quantifying variants that do not agree with the consensus lineage structure (Supplemental Fig. S9). We estimated the specificity and sensitivity of our branch SNV calls as a function of the quality threshold for accepting coincident variant calls, similar to the construction of a receiver operator characteristic (ROC) (Supplemental Fig. S10). For HT115 branch variant SNVs, the sensitivity and specificity were estimated to be 96.4% and 99.9% (HT115) and 91.9% and 99.9% (RPE1) based on the selected quality threshold of 0.99 applied to filter coincident SNVs when branch variants are called (Methods). The mutation spectra of the SNVs that do not agree with the consensus lineage structure differ from the spectrum for the lineage structure-concordant set for each cell line, consistent with the hypothesis that SNVs in disagreement with the called lineage structure represent errors that were correctly filtered out by our data analysis procedure (Supplemental Fig. S11). In contrast, mutation spectra of branch variant SNVs show strong similarity across sublineages, indicating the high specificity of these variant calls (Supplemental Fig. S12). In addition, we tested for bias affecting branch variant call sensitivity in relation to tree position. Individual branch variants have different numbers of subclones representing the variant; however, we did not detect any specific positional bias affecting branch variant call sensitivity (Supplemental Fig. S13).

### Lineage sequencing enables mutation rate determination with certainty in the generation number

We constructed a null statistical model for mutation accrual based on the assumptions that somatic mutation events were independent and that the number of mutations appearing in each daughter cell followed the same distribution (Poisson). We estimated the average mutation rate for HT115 cells as 173 SNVs per cell division with the 95% confidence interval (CI) [147,203] (using the model), which corresponds to a rate of 3.0 × 10^−8^, 95% CI [2.5 × 10^−8^, 3.5 × 10^−8^] SNV per bp per cell division. The error in this estimate is driven principally by the variant counting statistics since we have a reliable prior estimate of the number of generations over which mutations accrued in each lineage segment. At haploid loci, leaf variant data can also be used to independently estimate the mutation rate for SNVs at haploid loci with a allele fraction of one. These SNVs must occur prior to subclone expansion to appear clonal; otherwise, the SNV would be diluted in the population. We calculated the mutation rate on haploid sites for branch variants, 2.9 × 10^−8^ CI [1.3 × 10^−8^, 5.2 × 10^−8^] SNV/bp/division, and for leaf variants, 4.2 × 10^−8^ CI [2.2 × 10^−8^, 6.5 × 10^−8^] SNV/bp/division, at HT115 haploid sites and found rates similar to those calculated for diploid/triploid sites, suggesting that homology-directed repair (and any associated mutations) makes a minor impact in our HT115 experiment (Supplemental Fig. S14).

Consensus estimates of spontaneous mutation rates during nuclear DNA replication in normal cells are close to 5 × 10^−10^ SNV/bp/division (Blokzijl et al. 2016), indicating that the mutation rate we measured in HT115 cells is about two orders of magnitude higher than expected in normal cells and similar to previous estimates of the rate in cells with compromised POLE proofreading activity, although our HT115 line carries two normal *POLE* copies whereas many *POLE* mutant tumors are reported to carry one normal *POLE* copy (Billingsley et al. 2015). In contrast to the rapidly mutating HT115 cells, RPE1 cells showed an average mutation rate of 39 CI [27,52] SNVs per cell division, or 4.3 × 10^−9^ CI [2.9 × 10^−9^, 5.7 × 10^−9^] SNV/bp/division, seven times lower than observed in HT115 cells. For the most relevant estimation of the mutation rate ratio between the cell lines, we also compared rates after removing a subsignature of guanine oxidation (Cosmic signature 18, Supplemental Fig. S6) from the RPE1 data set that has previously been associated with in vitro culture and only rarely contributes many mutations under physiological conditions (Costello et al. 2013; Rouhani et al. 2016; Pilati et al. 2017; Zou et al. 2018). After removing the putative in vitro artifact from the RPE1 data set (HT115 had hardly any contribution from signature 18), we calculated the mutation rate difference between HT115 and RPE1 to be nearly 10-fold (HT115: 3.0 × 10^−8^ CI [2.5 × 10^−8^, 3.4 × 10^−8^], RPE1: 3.3 × 10^−9^ CI [2.0 × 10^−9^, 4.5 × 10^−9^]).

With time-to-division data available from the time-lapse imaging, our data structure enabled analysis of the dependence between mutation accumulation and generation time (Supplemental Fig. S15) but found no evidence for the accrual of mutations during inter-phase. This could be explained by limited statistical power in analysis of our small data sets, by the dominance of POLE substitution errors at replication, or by compensation of accruing mutations by more effective DNA repair in slow-cycling cells.

### Mutation rates vary across sublineages

**Figure 5.**
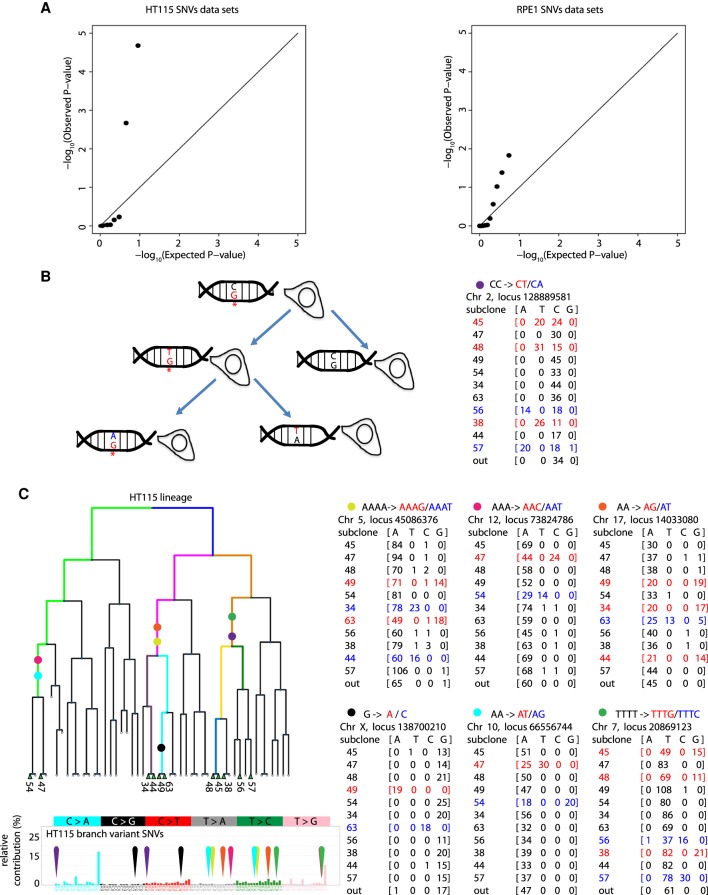
Intra-lineage heterogeneity in mutation rate and multiple mutation events. (*A*) Measured *P*-values from observed mutation counts are plotted vs. calculated theoretical (Poisson) *P*-values for the branch variant set for each sublineage to form quantile-quantile (QQ) plots (*P*-values for theoretical Poisson-distributed lineage-wise mutation count data versus observed data) for both HT115 (*left*) and RPE1 (*right*). The plotted points deviate strongly from the expected distribution (which would follow an *x* = *y* relationship) at both ends of the distribution, showing that the sublineages present in each data set cannot be plausibly modeled by a Poisson distribution based on independent mutations. (*B*) Schematic showing persistent lesion hypothesis for correlated same-site mutation. DNA lesions (marked as G*) that are not repaired during S-phase compel the DNA polymerase to replicate opposite lesion bases with a high probability of mutation. If the lesion has not been repaired before the next S-phase in the daughter cell carrying the lesion, an additional mutation at the identical genomic locus is likely to result. If the second mutation is different from the first, this process can be readily detected from lineage sequencing data. The example scheme represents the CC > CT and CC > CA mutations we detected at the Chromosome 2 locus 128889581 (marked with purple circle). (*C*) Seven multiple mutation events were found in the HT115 lineage. Read counts for each example are presented and marked with a colored symbol. The lineage segment where each example occurred is shown (with corresponding colors). None of these events overlap the most probable mutation types found in the *POLE* signature.

We next tested the presumption that mutations accrue according to a Poisson process (uniform probability per cell in time), or equivalently, that the cells in our lineage experiment growing under similar conditions at the same time would exhibit the same average mutation rate. We produced quantile-quantile (QQ) plots of *P*-value quantiles to compare the observed distribution of branch variant SNV counts across each lineage segment with the theoretical Poisson process model based on a constant rate of mutation. The QQ plots show poor concordance of the experimental data with the theoretical Poisson process model (Fig. 5A). This result indicates that mutations accrued across sublineages in HT115 and RPE1 experiments by a more heterogeneous process than expected from the Poisson model and that new models for mutation accrual with additional parameters to account for variable mutation rates are likely justified when analyzing mutational processes and interpreting mutation data.

### Multinucleotide variants exemplify variant nonindependence

Multinucleotide variants (MNVs) are known in the literature as somatic events where substitution mutations occur on neighboring nucleotides with higher frequency than would be expected by chance for independent variants (Rosenfeld et al. 2010; Besenbacher et al. 2016). We observe these events as strong enrichment (versus a random model) in closely linked mutations within the same sublineage (Supplemental Fig. S16, HT115 in A and RPE1 in B). The speculation that these linked variants arise at the same time as the concomitant occurrence of specific cluster events is supported by our finding that clustered events were consistently observed within sublineages but not across sublineages (Supplemental Fig. S16, HT115 in C and RPE1 in D). In addition, the accuracy and resolution of our data enable us to constrain the time window further and provide some evidence that the mutations in a cluster appear in the course of a single-cell cycle (example in Supplemental Fig. S16E). The SNVs from closely linked events also show a distinct mutation signature compared with other SNVs, indicating a different mechanism of origin (Supplemental Fig. S16, HT115 in part F and RPE1 in part G). This idea is also supported by the finding that, in RPE1, cells present a higher fraction of multinucleotide events (>2%) than HT115 cells (<0.5%), consistent with a mutational mechanism that is independent of POLE activity.

### Persistent lesions cause mutations in multiple cells

An extreme form of somatic event clustering are events where the genomic distance between two different SNVs is zero in two sister lineages. We observed 11 such instances where two different SNVs occurred at identical genomic positions in related sublineages (HT115: seven instances, Fig. 5C; RPE1: four instances, Supplemental Fig. S17). In our data set, it is extremely unlikely that two variants coincide by chance (*P* < 6.9 × 10^−5^ by estimating the frequency of chance overlaps of independent HT115 SNVs [leaf variants from different lineages]). Based on that null model, we could determine the chance to observe seven repeated events in the HT115 data set (*P* < 3 × 10^−5^) or four repeated events in the RPE1 data set (*P* < 1 × 10^−5^) by resampling (Supplemental Fig. S18A).

We hypothesize that these coincident mutation events result from DNA lesions that persist through multiple rounds of DNA replication and cause multiple, different substitution errors through translesion DNA synthesis opposite the same DNA lesion (Fig. 5B; Makridakis and Reichardt 2012; Sale et al. 2012; Sale 2013; Ziv et al. 2014; Bi 2015). Lesion persistence could result from common DNA lesions that escape repair by chance, other types of lesions that are repaired only slowly by cells, or damage-tolerant cell states (Geacintov and Broyde 2017). All the detected pairs of SNVs that occurred at identical genomic positions were found to be in related sublineages. Persistent lesions would necessarily generate overlapping mutations only within sublineages, not across sublineages. In contrast, random mutation processes should not be biased toward co-occurrence within the same sublineage. It is highly unlikely that independent random mutation pairs overlap only in related sublineages (HT115, 7/7, *P* < 6 × 10^−6^; RPE1, 4/4, *P* < 6.6 × 10^−4^) (Supplemental Fig. S18B).

These events can be effectively identified only when somatic variants are detected with high sensitivity and single-cell cycle resolution. The multiple mutation events also seem to show a different mutational spectrum than the complete branch variant sets, lending additional support to the idea that the multiple mutation phenomenon results from one or more unique molecular subprocesses. Linking multiple mutations to lesion persistence exemplifies how detailed analysis of high-quality variant data can be used to refine mutation signatures and parse mutation signatures for distinct biochemical processes.

## Discussion

Here, we describe lineage sequencing, a new genome-wide technique that utilizes knowledge of the cell lineage structure to reconstruct mutation events occurring during lineage formation with high resolution, high accuracy, high precision, and minimal bias. Lineage sequencing is based on popular short-read next-generation sequencing methods applied at typical shotgun sequencing coverage depth for each sequence library and utilizes joint variant calling across the set of sequence libraries informed by an estimate of the lineage structure. Our proof of concept implementation achieves partial resolution of individual cell cycles with neither whole-genome amplification nor polymerase chain reaction amplification steps and, as a result, produces sequence libraries with highly uniform and accurate coverage of the genome. By applying lineage sequencing, we measure the accumulation of SNVs by identifying >90% of SNVs that evolved during lineage formation and mapping these onto cells in the lineage with very high resolution (1–4 cell divisions, with little uncertainty in the number of cell divisions in each lineage segment where SNVs were assigned). Altogether, the method provides accurate and nearly complete estimates of cellular genotypes within the lineage, suggesting that lineage sequencing may emerge as a gold standard for somatic mutation quantification.

We were able to distinguish two types of SNV call sets: high-confidence branch variants that occur during lineage formation and leaf variants that occur in the last round of cell divisions or during subclonal culture. Leaf variants can be compared to variants identified by the existing “double bottleneck” methodology (Blokzijl et al. 2016; Szikriszt et al. 2016; Drost et al. 2017). Leaf variants cannot be assigned to a specific cell division in the lineage with high confidence and suffer from low specificity preventing precise quantification for mutation rate assessment. In contrast, our branch variant analysis shows improved performance over alternative methods by providing a nearly complete and highly accurate list of somatic variants that accumulate within a specific number of cell divisions.

Our motivation to develop lineage sequencing was to make measurement of mutagenesis more quantitative and more highly resolved in populations to enable a focus on mutational processes in addition to mutational outcomes and resulting effects on cell function. The common methods for measuring differences in mutation rate between cells require one or two bottleneck steps, with double-bottleneck protocols producing data sets with similar characteristics as our leaf variant data. In such approaches, including those we carried out to produce the leaf variant call sets, differences in the growth or death rates (which can be summarized versus a reference by a selection coefficient) affect the allele fraction at which mutations appear. High allele fraction variants can appear not only because the variant was present at the second bottleneck but also if a mutation appeared after the (second) bottleneck when the population size was still small or because a mutation appearing after the (second) bottleneck itself (or a linked variant) caused positive selection. These factors present technical challenges to variant calling and degrade confidence in the quantitative nature of mutation data. However, the branch variant set detected by lineage sequencing is not biased by early mutation events or selection downstream from the bottleneck.

Even so, lineage sequencing does have limitations that could affect results and in principle is subject to selection bias at two stages. The first is the choice of the lineages for analysis (or the cells with which to found lineages) from the overall population under study (first bottleneck). This can be addressed by making the sampling as random as possible and by obtaining data across lineage replicates. Second, bias could, in principle, arise when cells are subsampled from a particular lineage (second bottleneck). Ideally, all cells can be recovered and analyzed, but this may not be possible. In our experiments, sampling was limited by the viability of recovered cells, with survival rates observed to be ∼30% for HT115 and ∼65% for RPE1. When only some cells are analyzed, there is a potential for bias based on ease of recovery, culture or other means of genome amplification, amenability of a given sample to the sequencing technology used, or operator preference based on observed characteristics of sublineages or cells. Bias at the second bottleneck can be minimized by randomizing sublineage sampling. Here, we tested for bias in our sampled sublineages by comparing inter-division times observed by imaging in sublineages that were collected and that produced sequence data with those for which we were not able to produce sequence data. No evidence for significant bias based on inter-division time was found (Supplemental Fig. S2).

The use of the prior estimate of intersample relationships here is analogous to the use of known family relationships in human germline trio and quartet sequencing and provides high statistical power for variant detection (Roach et al. 2010) with a capability for assigning event timing. Since branch variants are clonal by definition, standard coverage depth (∼30×) supports extraordinary performance in their detection. In addition to the internal consistency of our branch variant analysis (Supplemental Figs. S5, S10, S12), these results are validated independently by agreement with the lineage structure established by time-lapse microscopy and the consistency of our aggregated de novo somatic variant sets with the expected mutation spectra, the expected impact of replication timing on mutation, and observed R-class asymmetry in *POLE* mutant samples. We found strong similarity of the HT115 whole-genome SNV spectrum with tumor samples sharing *POLE* deficiency and, among COSMIC exome mutation signatures, to the COSMIC *POLE* signature (Supplemental Fig. S6). These observations highlight the need for quantitative genome-wide signature assessment to take full advantage of somatic mutations to discriminate specific mechanisms of mutagenesis and DNA repair dynamics.

Our results show that mutations do not occur independently with uniform probability but rather are heterogeneous across the genome and across closely related cells, even when environmental conditions are uniform. We gained key insights into the short-term mutational dynamics of human cells, including indications that the measured mutation counts across lineage segments were overdispersed relative to a Poisson process, indicating that the mutation rate showed excess random variability across the lineage and/or across time on top of the mutation rate variability we observed across different genomic loci.

Multiple processes contribute mutations. In the samples sharing *POLE* deficiency, the dominant factor is likely the fraction of the mutated polymerase enzymes in the specific cell cycle, so variability in allele specific gene expression may add complexity that explains the observed overdispersion (Crowley et al. 2015). We also found evidence for two classes of nonindependent variant sets. We observed the first, previously described as multinucleotide variants (Rosenfeld et al. 2010; Besenbacher et al. 2016), and provide evidence that some of the linked mutation sets occur at the same time. In addition, we observed different mutations that occur at exactly the same genomic locus in related cells. Lesion persistence probably explains this and serves as an example of a biochemical mechanism capable of causing strong correlations among somatic mutations.

In principle, the observed time-to-replication for each cell in our imaging data can be used to determine whether the number of cell cycles, or alternatively, the elapsed time, is a better predictor of the number of somatic mutations, and further, to constrain likely mechanisms of mutagenesis in these cells. Our sample size provided insufficient power for this task, although the analysis illustrates the analysis framework enabled by our unique data structure (Supplemental Fig. S15). Where observations or perturbations are made of the cells during the development of the lineage, these can be directly related to genomic variants that appeared at the same times in the lineage. Targeting specific lineages in which intra-cellular events of interest were observed would enrich the variant set for mutations linked to these events (Supplemental Fig. S19). Much can be learned by studying real-time cellular phenotype data with linked genomic data from lineage sequencing, for example, the effects of different exogenous insults to cells, checkpoint activation, DNA repair capacity or stochastic dynamics, cell division defects, and more. Another feature of our approach is that the subclonal cultures can be grown to different population sizes and sampled at different times. This flexibility means that multiple genotyping methods with differing biomass requirements can be deployed optimally on the same subclone and that live cultures representing sublineages will typically be available for further experimentation.

Here, we showed two different ways that information about the lineage structure can be obtained prior to joint variant calling to support lineage sequencing: (1) by time-lapse microscopy; and (2) using raw SNVs from whole-genome sequence data (provided there is at least one detectable variant per cell to achieve full resolution of the lineage). While the lineage structures we derived from sequence data alone enabled recovery of the same set of relationships among the subclones as observed in the time-lapse microscopy, they lack independent information about the number of cell divisions and duration of each cell cycle that was available from the imaging data. Accurate lineage information is important for mutation rate estimation and could be uncertain from genomic data alone in cases where the number of mutations per generation is very small or when the sampling of sublineages is sparse. The latter is the case in our HT115 and RPE1 lineage experiments, and we use imaging data to ensure accurate cell division counts and precise mutation rate estimation (cf. the two dendrograms in Fig. 4C). Imaging capability also enables correlation of single-cell phenotypes with mutation data (as outlined in Supplemental Fig. S19). In principle, other approaches for tracking/estimating lineage structures could also support lineage sequencing (Woodworth et al. 2017), for example, approaches using recently reported self-editing genomic barcoding (McKenna et al. 2016; Frieda et al. 2017). With appropriate methods for cell sampling and subclonal culture, we expect that the lineage sequencing concept can be applied to study mutations that occur in solid tissues or whole organisms.

Lineage sequencing is able to overcome the consensus sequence error rate and provide higher power for genome-wide somatic event detection (Salk et al. 2018). Technical errors in standard sequencing approaches are introduced independently across subclones and are uncorrelated, resulting in dramatically lower false positive error rates in the branch variants calls where uncorrelated errors are excluded. In a simplistic view, the branch variant error probabilities are expected to fall by at least the square of the nominal consensus error rate, e.g., (10^−6^)^2^ = 10^−12^, since data from more than two independent samples contribute to the call. In addition to the samples providing evidence for the presence of the variant, the absence of variant reads in the remaining samples also supports the joint variant call. The extra statistical power lent by joint variant calling in lineage sequencing could conceivably support accurate and more sensitive mutation detection for direct single-cell readout that depends on noisy whole-genome amplification. Sampling strategies for lineage sequencing can be tailored to optimize statistical power for different estimation tasks; for example, sparser sampling of cells from the lineage could improve mutation rate determination at constant sequencing effort for cells with few mutations per generation.

The ability to check lineage sequencing variant call sets for self-consistent and independently predicted (e.g., from image data) variant relationships facilitates in-line data quality evaluation by enabling quantification of inconsistent candidate variants. In the examples reported here, the agreement of independent image-based and sequence-based lineage structures gave us high confidence that we determined correct lineage relationships among subclones. Data quality assessments can, in turn, be used to optimize the experimental and computational protocols (Supplemental Fig. S9).

In summary, lineage sequencing allows precise assessment of the rate and spectrum of somatic mutations with intra-lineage resolution as high as individual cell division events. Lineage sequencing can be combined with real-time phenotypic observation and/or perturbation to link cellular activity and responses with mutation events at the single-cell level. This detailed level of analysis enables the detection of individual biochemical events in cells and the parsing of mutation spectra with enhanced spectral/biochemical resolution. We imagine that lineage sequencing will be applied widely to study spontaneous and exposure- or therapy-associated mutational processes in remarkable detail. Such work would help identify the molecular mechanisms and biological consequences of somatic mutations broadly.

## Methods

### Cell culture and conditioned media preparation

Human HT115 epithelial colon carcinoma cells from the Cancer Cell Line Encyclopedia (CCLE) (Barretina et al. 2012) were maintained in high-glucose Dulbecco's modified Eagle's medium (DMEM, Life Technologies, Inc.) supplemented with 15% fetal bovine serum (Mediatech, #35-015-CV). The telomerase-immortalized RPE1 cells (ATCC) were maintained in DMEM/F12 medium (Thermo Fisher) with 10% fetal bovine serum. Conditioned medium for use with the microfluidic device was collected from the stock cell flask between 24 and 48 h after fresh medium was added. The medium was centrifuged (4700*g* for 5 min) and filtered (0.2 μm) before use.

### Microfluidic device

Hydrodynamic trap array devices were fabricated in silicon and glass as described previously (Kimmerling et al. 2016). Prior to cell culture in the device, the system was flushed with 10% bleach for 10 min for sterilization and cleaning, rinsed with water, and then flushed with conditioned cell culture medium overnight to fully rinse and prime the system for cell culture. Finally, devices were flushed with a 0.1% poly-L-lysine solution (Sigma) for 10 min to coat the channel surfaces and promote cell adhesion and growth, followed by a short wash of a few seconds with conditioned cell culture medium just before cell loading.

### Single-cell culture in the hydrodynamic trap array

A single-cell suspension was introduced at the downstream port of the system to load cells into the device (port P3 in Supplemental Fig. S1). In order to introduce cells into the device, the pressures P2 and P3 were increased equivalently (P2 = P3) and the pressure P1 was decreased (P2 > P1) such that there was equal flow from the downstream (P2, P3) to upstream (P1) ends of the bypass channels on either side of the hydrodynamic trap array. Once the dead volume of the system was purged and cells were within view in the device, single cells were manually loaded into each lane of the trap array. This cell loading was achieved by decreasing the pressure P2 relative to P3 (P2 < P3) to introduce reversed flow through the trap array (Supplemental Fig. S1). With this sustained flow into the trap array, the upstream pressure (P1) was periodically toggled between a higher pressure than P3 (P1 > P3) and atmospheric pressure (P1 < P3) to flow cells upstream and downstream near the entrance of the trap array to allow cells to slowly drift into each lane. Once a single cell had been loaded into each lane, the bypass channels were flushed with conditioned cell growth medium and the pressures were set for long-term culture such that media was constantly perfused along the bypass channels (P1 > P2, P3) and very slightly across the trap array (P2 > P3). This reverse-side loading approach ensured that only cells of interest entered the trap array and no cells or debris accumulated in the bypass channel on the left side of the device (channel connecting P1 and P2). Therefore, when cells were released from the trap array and flushed from the left bypass channel, there was significantly lower risk of collecting a contaminating cell or debris that had been caught in the channels or tubing during cell loading.

### Trypsinization for cellular reseeding or release

Following multigenerational growth in the device, cells were detached from the channel surfaces by introducing a solution of 0.25% trypsin and EDTA (Gibco). In order to achieve rapid fluidic exchange while minimizing shear stress on cells within the trap array, the pressures were set to have significant flow rate along the bypass channels (P1 ≫ P2, P3) while maintaining only slight flow across the trap lanes (P2 > P3). Fully dissociated cells were either reseeded in the device for continued culture or released one at a time for downstream collection and subclonal outgrowth. Cellular reseeding was carried out both across and within individual lanes of traps in the array. For instance, for longer-term lineage tracking, a single cell was loaded into one lane of the trap array and allowed to divide for two generations. After trypsin treatment, these cells were released into the left bypass channel (P3 > P2) where the pressures were set to have no flow (P1 = P2) and subsequently recaptured one cell per lane by maintaining slight flow across the array (P2 > P3) and periodically increasing the upstream pressure (P1 > P2) or setting it to atmospheric pressure (P1 < P2) to move cells along the length of the left bypass channel and position them for capture within each lane of the trap array. Following subsequent rounds of cell divisions, cells were reseeded within each trap lane by gently flowing forward across the trap array (P2 > P3) after detachment with trypsin. In both cases, the cellular detachment and recapture processes were recorded in order to maintain lineage information collected via time-lapse imaging. For single-cell collection downstream from the device, the pressures were set to have substantial flow rate along the bypass channels (P1 ≫ P2, P3) after detachment with trypsin—these pressures were set such that the volumetric flow rate along the bypass channel was ∼15 μL per minute. To release individual cells to the bypass channel, the flow direction was periodically reversed (P3 > P2) until a cell reached the bypass channel at which point flow into the traps was re-established (P2 > P3) to ensure no other cells were released. Each cell was flushed for 30 seconds (∼7.5 μL total volume, in order to clear the dead volume of the system) and collected directly into 70 μL of conditioned cell culture medium in a glass-bottom 384-well plate for continued subclonal outgrowth in a standard tissue culture incubator (37°C, 5% CO_2_).

### Cell identity tracking and lineage reconstruction by time-lapse image analysis

Time-lapse imaging was conducted with a custom LabView program (National Instruments), which drove a TTL-triggered white LED light-source (ThorLabs) for illumination, as well as two automated stages (Newport), which traversed the *x*- and *y*-axes to capture multiple fields of view for each frame. Images were taken every 3 min (using 10×-magnification lens). We experienced one rare case of a longer gap between images; however, cell identities were preserved at all times and tracking ability was not affected. Lineage structure and time to division measurements were determined by manually tracking the recorded image series using ImageJ software (example image series shown in Supplemental Movie MS1). The trypsin release and reseeding and single-cell release image series were captured continuously using a lower magnification (4×) lens that captured the entire device image in a single field of view. These image series were analyzed with the assistance of custom Python code based on the OpenCV library package that pre-analyzed the movies and marked each cell with a different color to ease human analysis, which was performed with iMovie software (Apple, Inc.).

### Cell growth measurements

Single cells released into separate wells on a glass-bottom plate for subclonal culture were immediately imaged upon collection to validate the presence of a single cell per well. The growth of subclones was monitored every 24–48 h, and fresh conditioned medium was introduced every 48 h. For HT115 lineage, we isolated 37 cells (out of 45 cells in the channel), of which 11 grew as subclonal cultures and were processed for sequencing. For RPE1, we isolated 22 single cells (out of 26 cells in the channel), of which 15 grew as subclonal cultures. Thirteen of these subclonal cultures were processed for sequencing.

### gDNA extraction and library construction

Colonies were grown to at least 10^6^ cells. Genomic DNA was extracted from each sample using the QIAamp DNA Mini kit (Qiagen). PCR-free library construction was performed by the Genomics Platform at the Broad Institute using their standard process. All sample information and tracking was performed by automated LIMS messaging. Samples underwent fragmentation by means of acoustic shearing using a Covaris focused ultrasound shearing instrument to provide fragments of ∼385 bp. Following fragmentation, size selection was performed using a SPRI cleanup. Library preparation was performed using a commercially available kit (product KK8202, KAPA Biosystems) that entailed palindromic forked adapters with unique 8-base index sequences embedded within the adapter (the DNA oligonucleotide adapters were purchased from IDT). Following library construction, each library was quantified using quantitative PCR (qPCR; KAPA Library Quantification kit [ABI Prism] from KAPA Biosystems). This qPCR quantification assay was automated using Agilent's Bravo liquid handling platform. Based on the qPCR quantification, libraries were normalized to 1.7 nanomolar. Library samples were then pooled into groups of 24 samples, and the 24-plex pools were once again quantified by qPCR. Library pools were then combined with HiSeq X Cluster Amp Mix 1, 2, and 3 in a tube strip using the Hamilton Starlet Liquid Handling system. Cluster amplification of the templates was performed according to Illumina's protocol using the cBot instrument (Illumina). Clustered flow cells were subjected to shotgun sequencing on HiSeq X to ∼35-fold coverage of the genome using proprietary sequencing by synthesis (SBS) reagents (Illumina HiSeq X) and analyzed using RTA2.

### Primary analysis of genomic data

Sequence read alignment, data aggregation, preliminary production analysis, and quality control proceeds after sequencing using the automated Picard pipeline (Broad Institute Genomics Platform, http://picard.sourceforge.net/). The Picard pipeline produces high quality recalibrated sample level BAM files using the following procedure. Reads were extracted from sequencing instruments and aligned using BWA against the hg19 reference. Duplicate reads were marked for downstream interpretation in the analysis pipeline. Reads around known indel sites were realigned to produce improved alignments. Quality scores were recalibrated using the GATK base quality score recalibrator to increase the accuracy of reported base quality scores. Cell line identity was verified against reference genotype fingerprints. Data were aggregated per sample in BAM format including base calls, quality scores, and alignment data. Finally, summary metrics were generated to allow quality assessment.

### Copy number variation (CNV) analysis

Copy number variation (CNV) analysis for each cell line (Supplemental Fig. S4) was carried out by the procedure outlined below and showed that HT115 cells were largely diploid as expected. However, CNV analysis showed that our RPE1 cells were predominantly triploid, which might be related to genomic instability caused by telomerase/telomere dysfunction (Garbe et al. 2014), as the RPE1 line was originally immortalized by telomerase expression. For CNV analysis, reads were counted for each sample in 10,000-base bins using the GATK 4 Tool SparkGenomeReadCounts function and divided by the median bin coverage. By exploiting the fact that each sample came from a pure subclonal population, coverage was scaled by a factor of α where α minimized the objective sum{bins}(abs(α coverage(bin) − nearest integer(α coverage(bin)))). This had the result of scaling coverage scores to line up with integers as well as possible. The absolute (but noisy) coverage level values were entered into a Hidden Markov Model (HMM) with integer copy number states 0, 1, 2, 3, 4, 5. This HMM learned transition matrix elements between states via an expectation–maximization (EM) algorithm. The observed coverage was modeled as a Cauchy distribution, centered on the integer copy number values, and the model learned the width parameter of this emission distribution via the EM algorithm. The parameters were updated by an M step consisting of numerical optimization while the E step consisted of obtaining posteriors from the forward-backward algorithm. Once the model had converged, the Viterbi algorithm was used to obtain segments of constant copy number. With these calculations we could estimate the total size of the genome in each of our cell lines, finding 5.7 × 10^9^ bp for HT115 and 9.2 × 10^9^ bp for RPE1.

### Single nucleotide variant (SNV) analysis

Raw single nucleotide variants were identified using MuTect1 (Cibulskis et al. 2013), MutTect2, and Strelka (Saunders et al. 2012) within the “Firehose” pipeline, developed at the Broad Institute (www.broadinstitute.org/cancer/cga). The variant callers were run for every pair of subclones twice, such that each sample acted as a “tumor” sample in one run for a subclone pair, and as a “normal” sample in another run of the same subclone pair. The SNV lists from all samples were combined and de-duplicated using a custom Python script (Supplemental File S5).

### Lineage sequencing analysis pipeline

Raw SNVs for each lineage experiment were extracted from the combined SNV caller output list. Candidate coincident SNVs, identical SNVs appearing at matching genomic loci in two or more but not all subclones from a lineage experiment, were identified. DNA base counts were extracted for all subclones at the locus for each coincident SNV. SNVs that contained low coverage (<5 reads) of alternate alleles (compared with the reference) in one of the variant samples were not pursued further. Coincident SNVs were called where the quality of coincident SNV classification was assessed by calculating the probability for each sample to belong to its consensus-assigned group (‘reference’ or ‘alternative’) considering the base content in the read alignment at the locus in question for each sample. The probability was calculated using a binomial distribution test for each of the samples. Assuming each sample can belong to the assigned reference or variant group (H0) or to the nonassigned group (H1). The calculated probability serves a heuristic score to rank the quality of coincident SNV groups.

H0: P = P1, the probability weight that the sample belongs to group1, the assigned reference or variant group.

H1: P = P2, the probability weight that the sample belongs to group 2, a different group than assigned.

$$P1=P({\mathrm{alt}}_{\mathrm{allele}}=x\;\in \mathrm{group}\;1)\;=\left(\begin{array}{c} \mathrm{\#alt}\\ \mathrm{total\_nucleotides} \end{array}\right)\;P1^{\#\mathrm{alt}}{\left(1-P1\right)}^{\#!\mathrm{alt}},$$

$$P2=P({\mathrm{alt}}_{\mathrm{allele}}=x\;\in \mathrm{group}\;2)=\left(\begin{array}{c} \mathrm{\#alt}\\ \mathrm{total\_nucleotides} \end{array}\right)\;P2^{\#\mathrm{alt}}{\left(1-P2\right)}^{\#!\mathrm{alt}},$$

$$P(H0|\mathrm{alternative}\;\mathrm{allele}=x)=\frac{P1}{P1+P2}.$$

For each classified SNV we calculated this probability for every sample (P1/(P1 + P2)) and chose the minimal probability to represent the quality of this coincident classification option to the SNV. We filtered out all SNVs with quality of this coincident classification less than 0.99 (Supplemental Fig. S9).

Two approaches were used to obtain a prior estimate of the lineage structure for use in calling the final branch variant sets:

Method 1: In the “optical tracking → lineage → called variants” approach, the lineage structure was determined by analysis of time-lapse imaging of cells in the microfluidic trap array device. This lineage structure was subsequently used for calling branch variants. SNVs were grouped as coincident SNVs and branch variants called by evaluating the coincident SNV quality score for the highest scoring group of subclones over all the groups of subclones consistent with the lineage structure determined by time-lapse imaging, including the option that an SNV is only truly present in a single subclone and represents a leaf variant.

$$\mathrm{Coincident}\mathrm{SNV}\mathrm{quality}\mathrm{score}=\left(\mathrm{Max}P_{\mathrm{clusters}}\left(\mathrm{Min}P_{\mathrm{sample}}\left(\frac{P1}{P1+P2}\right)\right)\right).$$

Method 2: In the “raw variants → lineage → called variants” approach, we used the shotgun sequence data to estimate the most likely lineage structure in each lineage experiment. This was accomplished by identifying coincident SNVs by analysis of raw SNV calls from the subclones generated by the variant caller (Supplemental File S5). Subclones sharing the same SNVs were grouped without restrictions by hierarchical clustering, and for each coincident SNV, the quality score was calculated as: Coincident SNV quality score = (Min *P*_samples_ (P1/(P1 + P2))). The frequency of coincident SNVs for each group of subclones was tabulated and ranked to generate the plots in Figure 4B. For the experiments with both cell lines, the groups of subclones sharing a large number of high-scoring coincident SNVs nested in such a way as to indicate a single lineage structure for each of the experiments. This lineage structure was subsequently used for calling branch variants. The prior estimated lineage structure was used to filter the high-quality coincident SNV list for coincident SNVs that were consistent with the lineage structure and produce the final branch variant call set.

Filtering the loss-of-heterozygosity (LOH) variants: LOH can detect SNVs resulting from deletions or duplications of parts of chromosomes in an accurate and efficient way and further suppress false-positive SNVs (Roberts et al. 2013). MuTect has such a capability when applied in the conventional way comparing tumor and normal sample pairs. However, in our case we perform analysis across variable numbers of samples, so we needed to identify and remove LOH events in a separate step. LOH SNVs are defined as SNVs where the alternative allele fraction > 0.85 AND the reference allele fraction < 0.85.

### Classifying SNVs in the first cell division of the lineage

By grouping the samples in each branch variant SNV and comparing to the base call at the same locus in a sister lineage, we could determine which was the reference allele and which was the alternative and calculate the variant allele fraction without depending on an external reference allele. However, for the first cell division, no such sister lineage exists within our data set, so we do need an external reference to identify the reference genotype for the cell that founded each lineage experiment. In the HT115 lineage, this involved the split across subclones {{47,54}; {34,44,49,63,48, 45,38,56,57}} and in the RPE1 lineage, the split across subclones {{46,39,34,37,38}; {28,32,36,24,27,44,23,22}}. In order to resolve this issue, we compared each branch variant SNV in these groups to the reference genome (hg19) for RPE1 cells and assigned alleles matching hg19 as a reference. We were less confident in using hg19 to identify reference alleles in the fast-mutating HT115 cells, so instead we used base calls from an additional HT115 subclone we sequenced from a different HT115 lineage experiment (seeded from the same HT115 cell stock) to identify reference alleles at the top of the HT115 lineage presented in this study. An additional consistency check was performed to evaluate if the correct alleles were assigned as reference after the first generation in the lineage by verifying that the majority of the SNVs represent homozygote to heterozygous changes as expected, which was indeed found to be the case.

### Cell line authentication

Cell identity was authenticated by the Broad Genomics Platform for HT115 using previously stored fingerprint genotypes. The fingerprint consists of the genotype at 82 loci from the query sample which were compared against fingerprints of all the Cancer Cell Line Encyclopedia cell lines using the farthest neighbor graph (FNG) algorithm. The highest correlation (0.83) was found between our HT115 sample and the CCLE HT115 sample. For RPE1 (which is not a CCLE cell line), we used the ATCC short tandem repeat (STR)-based authentication service. The STR profile of our RPE1 sample validated with 100% similarity to hTERT RPE1.

### POLE tumor WGS data sets

We assembled a collection of 10 whole-genome tumor data sets that were published and annotated with POLE coding mutations and mutator phenotype from the Cancer Genome Atlas (TCGA; dbGAP: phs000178.v1.p1) (Haradhvala et al. 2016).

### Mutation spectrum calculation

Mutation spectra were defined using standard approaches and reported in the standard format (Alexandrov et al. 2013b). Each SNV was classified into one of six subtypes—C:G > A:T, C:G > G:C, C:G > T:A, T:A > A:T, T:A > C:G, and T:A > G:C—and further refined by including the sequence context of each mutated base (one base 3′ and one base 5′). For example, a C:G > T:A mutation can be characterized as TpCpG > TpTpG (mutated base underlined and presented as the pyrimidine partner of the mutated base pair) generating 96 possible mutation types (six types of substitution × four types of 5′ base × four types of 3′ base). We compared the mutation spectra from our HT115 and RPE1 branch variant and leaf variant SNV call sets with patterns extracted from POLE mutant clinical and cell line (ATCC) samples (Fig. 3A; Supplemental Table S1). The similarity between two mutation patterns A and B, defined as a nonnegative vector with 96 mutation types, was computed by cosine similarity:

$$sim(A,B)=\;\frac{\sum_{i=1}^{n}A_{i}B_{i}}{\sqrt{\sum_{i=1}^{n}A_{i}^{2}}\sqrt{\sum_{i=1}^{n}B_{i}^{2}}}\;.$$

A larger cosine similarity value indicates mutation spectra are more similar to one another. Ninety-five percent confidence intervals were calculated by bootstrapping the SNV list 10^4^ times.

### Replication timing analysis

DNA replication timing for HT115 cells was measured according to a previously described method (Koren et al. 2012; Supplemental Table S4). Briefly, 50 million cells were fixed with EtOH, treated with RNase A, and stained with propidium iodide (PI) for DNA content. G1 and S phase cells were sorted using the FACSAria III cell sorter (BD; 1 million cells per fraction), and genomic DNA was extracted and whole-genome sequenced. Replication timing was calculated by counting the number of S phase reads in consecutive windows containing 200 G1 reads along each chromosome, filtering outlier data points, and smoothing the data with a cubic smoothing spline. We arbitrarily divided the genome into early- (≥60), intermediate- (>33 and <60), and late- (≤33) replicating bins. The 95% confidence intervals (presented as error bars) were calculated by bootstrapping the SNV list 10^4^ times. Replication timing data for the RPE1 data set were obtained from a general previously published data set (Koren et al. 2012).

### Branch variant SNV occurrence in genes and exons

Genomic regions consisting of genes, exons, and introns were determined from RefSeq gene tables (Pruitt et al. 2007). The total fraction of these regions in the genome were counted and normalized with attention to the locus-wise CNV calls. The log_2_(observed/expected) ratios of the branch variant SNV count values in each genomic region type were calculated. One-tailed binomial tests were performed to calculate the statistical significance of deviations by the observed counts from the expected number of mutations (based on the average genome-wide count for each lineage experiment and the size of each genomic region type considered) using binomial statistics (custom Python code); *P* < 0.01 was considered significant. The 95% confidence intervals (presented as error bars) were calculated by bootstrapping the SNV list 10^4^ times, recalculating results for each list, and determining the 0.025th and 0.975th quantile values.

### Analysis of replication-class (R-class) asymmetry

Left- and right-replicating regions were calculated from replication timing (Supplemental Table S4) measurements as described previously (Haradhvala et al. 2016). Regions with 0.1 < slope < 0.3 units/interval were designated right-replicating, and regions with −0.3 < slope < −0.1 were designated left-replicating. In order to determine the reference strand asymmetries (a control for other types of asymmetry), each of the 12 possible substitutions with respect to the genomic reference strand (six base-pair changes × two orientations) were counted and normalized by the number of corresponding bases in the genome to measure mutations/Mb. Rates of complementary mutations (e.g., C > A and G > T) were then compared. To measure replicative strand asymmetries, all SNVs were counted with respect to the leading strand template as described (Haradhvala et al. 2016) using the left- and right-replicating regions defined above. For example, C > A mutations in the leading strand reference are considered to be genomic reference strand C > A mutations in left-replicating regions and genomic reference strand G > T mutations in right-replicating regions. Mutation rates were again calculated by normalizing for the number of corresponding bases in the genome within our intervals with defined replication direction, and the complementary mutations were compared. Error bars for mutation rates represent a 95% confidence interval for the underlying binomial probability of a given base being mutated, calculated from the beta distribution parameterized as Beta (*n* + 1, *N*−*n* + 1), where *n* is the number of a given mutation and *N* is the size of the genomic territory of the mutated base. *P*-values represent the binomial probability of seeing a given distribution of complementary mutations, assuming the probability of a given mutation is determined solely by the base composition within an interval (e.g., the probability of seeing a C > A instead of a G > T is the proportion of C:G base-pairs with a C on the strand of reference. This will be very near a value of 0.5). Strand asymmetries were calculated as log_2_ of the ratio of complementary mutation counts. Error bars represent a 95% confidence interval on the log_2_ quotient of the underlying binomial probabilities above. These confidence intervals were determined empirically by taking 1000 pairs of samples from the beta distribution above for both complementary mutation sets, taking the log_2_ quotient of each sampled pair, and determining the 0.025th and 0.975th quantiles of the resulting distribution.

### Calculation of branch variant SNV sensitivity and specificity in lineage sequencing

Due to the lack of a sufficiently accurate independent validation method (Schmitt et al. 2012; Salk et al. 2018), we estimated the sensitivity and specificity of lineage sequencing by checking for the internal consistency of the structured lineage sequencing data and its consistency with the image-based tracking data. False-positive and false-negative SNV call rates were estimated by applying the “raw variants → lineage → called variants” approach to generate calls and analyzing the results in the context of the optical cell tracking-based lineage structure. The consensus lineage structure estimated from the genomic data agreed with the lineage structure determined by time-lapse imaging and was assumed to be the true lineage structure for this analysis. In the following, coincident SNVs were considered to be true positives when in agreement with the consensus lineage structure and false positives when conflicting with the consensus lineage structure.

Next, we prepared a scrambled version of our data set where subclone labels were randomly scrambled for each SNV call, and false-positive SNV call rates were calculated by summing the count of coincident SNVs in the scrambled data set that agreed with the consensus lineage. The data were scrambled by building a list of 10,000 alternative scrambling patterns. Each pattern was checked to verify that each real subclone set representing the known true lineage structure was indeed disrupted by the scrambling operation and would not create bias. For each SNV, we randomly picked a scrambling pattern from the list and reassigned SNVs to the new subclone identities. The scrambled data set was reanalyzed and the false-positive coincident SNV rate found to be five SNVs for the HT115 lineage experiment and 24 SNVs for the RPE1 lineage experiment, where the quality threshold for accepting coincident SNVs was set to ≥0.99 (Supplemental Fig. S9).

False-negative SNV counts were estimated by counting the number of true SNVs that were filtered out as a result of their failure to surpass the required quality threshold for accepting coincident SNVs. We recognize that these “missing” SNVs could likely be imputed to improve the sensitivity of the branch variant SNV call sets, but we have not performed such an imputation in this study and seek here to report the “raw” false-negative rate. We start with the true-positive branch variant SNVs, then query subclones where the consensus lineage structure would have predicted the same SNV to exist but none was called due to the “missing” SNV falling short of the quality threshold applied (0.99). From the counts of these “missing” SNVs, we subtract the estimated false positive counts separately estimated at this quality threshold to arrive at the estimated false negative SNV count

FN=[#branch_variants<threshold]−[#scrambled_branch_variants<threshold].

With True Negative (TN), False Positive (FP), True Positive (TP), and False Negative (FN) counts, we could estimate the specificity and sensitivity of lineage sequencing for the HT115 and RPE1 lineage sequencing data sets with the coincident SNV quality threshold at 0.99

Specificity=TN/(TN+FP)=0.999(HT115andRPE1),

Sensitivity=TP/(TP+FN)=0.964(HT115)and0.918(RPE1).

We then estimated the sensitivity and specificity of SNV calls using the above estimation approach as a function of the coincident SNV quality threshold value to produce the plots in Supplemental Figure S10. An additional possible source of false-negative branch variant SNV calls are coincident SNV calls that surpass the quality threshold but only partially agree with the consensus lineage structure and were excluded—for example, coincident SNVs that agree with two out of three samples in a clade of the consensus lineage structure. These cases were not found to be higher than expected based on background raw SNV noise.

### Generating lineage dendrograms from raw sublineage SNV calls

Dendrograms representing cell lineage relations between samples were measured by counting the number of high-quality coincident SNVs between every pair of samples normalized by the total number of high-quality coincident SNVs. One minus this matrix of similarity scores results in a matrix of distance scores between every pair of samples. This pairwise distance matrix was used to render dendrograms using the MATLAB seqlinkage tool.

### Removing putative in vitro artifacts

To avoid studying in vitro artifacts (Zou et al. 2018), we removed the oxidation-associated spectrum from the data by de-convoluting a subsignature of guanine oxidation (COSMIC signature 18) from the branch variant call sets. We performed a projection approach to determine the sample-specific attributions of 30 COSMIC mutation signatures by modifying “SignatureAnalyzer” (http://archive.broadinstitute.org/cancer/cga/msp) (Kasar et al. 2015; Kim et al. 2016). More specifically, the projection was done by minimizing the Kulbeck-Leibler divergence between the mutation count matrix, *X* (96 × *N*), where *N* is the number of possible sublineages (branches and leafs subclones) from the two cell lines, and a product of the signature-loading matrix *W* (96 × 30) and the activity-loading matrix *H* (30 × *N*). During the optimization, the signature-loading matrix *W*, comprised of the normalized signature profiles of 30 COSMIC signatures, was strictly frozen and the activity-loading matrix *H* was iteratively refined through the multiplication update scheme to best approximate the mutation count matrix *X ∼ WH*. The resulting row vectors in *H* represent a de-convoluted signature activity across samples (Abeshouse et al. 2017). The contribution of COSMIC signature 18 was subtracted from all branch variant counts, and mutation rates were recalculated.

### Statistical analysis of mutation rate and quantile-quantile plotting

The counts of branch variants (Fig. 2) were assumed to follow independent Poisson distributions (Gelman et al. 2013) with a constant rate proportional to the number of cell divisions (=generations). Let *y*_*i*_ be the number of mutations per branch, where *y*_*i*_|λ ∼ Poisson(λ × *n*_*i*_), where *n*_*i*_ is the number of generations per branch.

We assume the noninformative prior distribution of λ|gamma(α,β) with α = 0, β = 0. So, the posterior distribution is then

$$\lambda |y∼gamma\;(\alpha +n\times y_{\mathrm{mean}},\;\beta +n).$$

We then generated the distribution of λ, which resemble the number of mutations per generation and determined as well as the 0.025th and 0.975th quantiles of the resulting distribution. These counts were normalized by dividing out the total size of the genome taking into account the regional copy number variation of each line (for HT115, total bp of 5.7 × 10^9^, and for RPE1, total bp of 9.2 × 10^9^) to obtain the mutation rate with units of SNV/bp/cell division.

The *P*-value calculations for branch variant counts in each lineage segment were done by simulation of the Poisson model 10^6^ times with dependence on the segment length. The quantile-quantile plot was produced by comparing the log_10_ of the sorted observed *P*-values against the log_10_ of the sorted expected *P*-values.

### Haploid mutation count validation

The full list of branch and leaf variants in the HT115 lineage experiment was reduced to those SNVs that occur in haploid regions by filtering allele fraction SNVs where both the reference allele fraction was >0.9 and the alternative allele fraction was also >0.9. Leaf variant SNVs in this set are likely to be true SNVs that occurred in the last generation of the lineage experiment. The total length of haploid regions was calculated in consideration of the measured copy number and totaled 3.05 × 10^8^ bp. We estimated the mutation rate in these regions. We then simulated the Poisson counting statistics as described before 10^6^ times and determined the 0.025th and 0.975th quantiles of the resulting distribution to establish confidence intervals.

### Detection of multinucleotide variants

The distances between variants were measured and compared to the expected distances between variants that were generated randomly assuming random uniform spacing of mutations across the genome. We simulated random sets of 12,662 SNVs for HT115 (branch and leaf variants) and 1985 SNVs for RPE1 (branch and leaf variants) 10^3^ times and calculated distance distributions. Then, for each SNV we calculated the distance to the closest SNV from the same lineage from (1) all other detected SNVs, or (2) all other SNVs except SNVs that shared between the same group of subsets.

### Detection of different SNVs that occurred at identical genomic positions

In order to identify SNVs that occurred at identical genomic positions, we modified the “raw variants → lineage → called variants” approach by allowing initial clustering into three groups (‘reference,’ ‘alternative 1,’ or ‘alternative 2’). Initial coincident SNVs were then determined by hierarchical clustering of three groups for each SNV (Scipy hierarchy fcluster). The center of every cluster was calculated and the quality of coincident SNV classification was calculated similarly as above, by the probability of each sample to belong to its consensus-assigned group

$$P(\mathrm{sample}\in \mathrm{assigned}\mathrm{group})=\frac{P1}{P1+P2+P3}.$$

The final quality score of the multiple event coincident mutations was determined by taking the minimum probability sample as describing each sample

$$\mathrm{Coincident}\;\mathrm{SNV}\;\mathrm{quality}\;\mathrm{score}=\left(\mathrm{Min}P_{\mathrm{sample}}\left(\frac{P1}{P1+P2+P3}\right)\right).$$

### Calculating the probability of getting two variants coinciding in the same locus in our data set

Initially, we estimated the probability of multiple variants coinciding at the same genomic locus for uniform probability along the whole genome by simulating (10^4^ times) the chance of getting more than one repeated event out of 12,662 total events in HT115 (all branch and leaf variants), or out of 1985 total events in RPE1, which provide a low probability for a repeated event *P* < 2 × 10^−6^. However, the assumption of uniform probability over the genome is not accurate (Fig. 2C,D; Supplemental Figs. S7, S8) and underestimates the probability of mutation coincidence. For a more accurate estimate, we used data available from an additional HT115 subclone we sequenced from a different HT115 lineage experiment, seeded from the same HT115 cell stock (subclone ‘out’) (Fig. 2A). Subclone ‘out’ has mutations that are independent of the specific mutations in our HT115 lineage experiment but have the same underlying distribution. We could test for overlaps with the leaf variant set from our lineage experiment; two overlaps were found. To determine the number of effective comparisons (denominator), we compared subclone ‘out’ with the estimated genotype of the cell that founded the lineage experiment and found about 18,901 SNVs that separate sample ‘out’ from our lineage ancestor + 9880 more leaf variants that accumulate separately in the lineage. We searched for overlapping mutations among the samples and found two overlaps from a set of 28,781 independent SNVs. This provides a rate of overlap from representative null data we can use to determine a *P*-value for the overlap rate we actually observed in the HT115 lineage experiment (seven overlaps out of 12,662 total intra-lineage variants). After running the simulations (10^5^ times) we estimated the probability to observe several repeated events (Supplemental Fig. S17A): seven cases in HT115 (*P* < 3 × 10^−5^) and for four cases in RPE1 (*P* < 1 × 10^−5^).

### Probability that overlapping SNV pairs co-occur within sublineages

In our results, all the detected pairs of SNVs which occurred at identical genomic positions occurred within a given sublineage. We tested the likelihood that independent pairs of SNVs occurring at identical genomic positions will fall within the same sublineage. We simulated two independent events at a time using the probability of getting mutation from each specific subset of samples. We simulated 5 × 10^5^ instances and for each chose randomly two possible branch/leaf variants from our call sets (four times for RPE1 and seven times for HT115). For every set of pairs, we measured the fraction of pairs that were congruent with the lineage structure and plotted the resulting distribution of observed fractions for RPE1 and for HT115. In the simulation, 1–3 out of 500,000 cases gave this result for HT115 (seven events), indicating a *P*-value less than *P* < 6 × 10^−6^. *P* < 6.6 × 10^−4^ was found for four events in RPE1 (Supplemental Fig. S18B).

## Data access

All sequencing data produced in this study (aligned to hg19[GRCh37]) have been submitted to the NCBI Sequence Read Archive (SRA; https://www.ncbi.nlm.nih.gov/sra/) under accession number SRP159787. Custom Python source code is available in Supplemental File S5 and on GitHub (https://github.com/yehudabrody/Lineage-sequencing---proof-of-concept).

## Competing interest statement

The Broad Institute and MIT may seek to commercialize aspects of this work, and related applications for intellectual property have been filed.
